# Growth form, regeneration mode, and vegetation type explain leaf trait variability at the species and community levels in Mediterranean woody vegetation

**DOI:** 10.1002/ece3.11145

**Published:** 2024-03-11

**Authors:** İrem Tüfekcioğlu, Çağatay Tavşanoğlu

**Affiliations:** ^1^ Institute of Science Hacettepe University Ankara Turkey; ^2^ Division of Ecology, Department of Biology Hacettepe University Ankara Turkey

**Keywords:** growth form, Mediterranean Basin, plant traits, regeneration strategy, resprouting ability, trait variability

## Abstract

Leaf traits are good indicators of ecosystem functioning and plant adaptations to environmental conditions. We examined whether leaf trait variability at species and community levels in Mediterranean woody vegetation is explained by growth form, regeneration mode, and vegetation type. We studied several plant communities across five vegetation types – semi‐closed forest, open forest, closed shrubland, open shrubland, and scrubland – in southwestern Anatolia, Türkiye. Using linear mixed models, community‐weighted trait means, and principal component analysis, we tested how much variability in three leaf traits (specific leaf area, leaf thickness, and leaf area) is accounted for species, growth form, regeneration mode, and vegetation type. Despite a large amount of leaf trait variability both within‐ and among‐species existed, functional groups still accounted for a significant part of this variability. Resprouters had higher SLA and leaf area and lower leaf thickness than non‐resprouters. However, further functional separation in regeneration mode, by considering the propagule‐persistence trait and the seed bank locality, explained leaf trait variability better than only resprouting ability. Although no consistent pattern was observed in three leaf traits in the growth form, we found evidence for the difference in SLA and leaf thickness between shrubs and large shrubs, and subshrubs had smaller leaves than other growth forms. Vegetation type also accounted for a substantial amount of leaf trait variability. Specifically, plant communities in closed habitats had larger leaf area than open ones, and those in scrublands had higher SLA, lower leaf thickness, and lower leaf area than other vegetation types. Climate and phylogeny had limited contribution to the results obtained, with the exception of a significant phylogenetic effect in explaining the differences in SLA between resprouters and non‐resprouters. Our results suggest that multiple drivers are responsible for shaping plant trait variability in Mediterranean plant communities, including growth form, regeneration mode, and vegetation type.

## Graphical abstract

We found a large amount of leaf trait variability within and among species in Mediterranean plant communities. However, functional groups (resprouting ability, regeneration strategy, growth form) and vegetation type still accounted for a significant part of this variability. Our results suggest that multiple drivers are responsible for shaping plant trait variability in Mediterranean plant communities, including growth form, regeneration mode, and vegetation type.

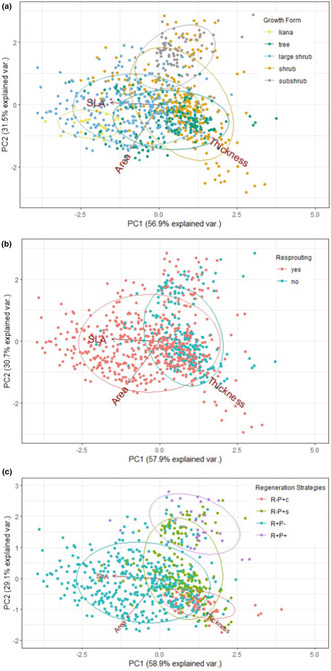

## Özet

Yaprak karakterleri ekosistem fonksiyonunun ve bitkilerin çevresel koşullara uyarlanımlarına dair iyi göstergelerdir. Bu çalışmada, Akdeniz orman vejetasyonundaki tür ve komünite seviyesinde yaprak karakter değişkenliğinin, büyüme şekli, rejenerasyon modu ve vejetasyon tipi tarafından açıklanıp açıklanmadığını inceledik. Türkiye'nin güneybatısında, beş farklı vejetasyon tipinde ‐ yarı kapalı orman, açık orman, kapalı çalılık, açık çalılık ve bodur çalılık ‐ birçok bitki komünitesini inceledik. Doğrusal karma modeller, komünite ağırlıklı karakter ortalamaları ve temel bileşen analizi kullanarak, üç yaprak karakterinin (özgül yaprak alanı, yaprak kalınlığı ve yaprak alanı) değişkenliğinin ne kadarının tür, büyüme şekli, rejenerasyon modu ve vejetasyon tipi tarafından açıklandığını sınadık. Hem tür içi hem de türler arası büyük miktarda yaprak karakter değişkenliği olmasına rağmen, fonksiyonel gruplar bu değişkenliğin önemli bir kısmını açıklamıştır. Yeniden sürgün veren türlerin SLA ve yaprak alanı daha yüksek, yaprak kalınlıkları ise yeniden sürgün veremeyenlere göre daha düşük olarak bulunmuştur. Ancak, rejenerasyon modunda daha fazla fonksiyonel ayrıma gidilmesi, yalnızca yeniden sürgün verme yeteneğinden ziyade, tohumla yerleşebilme özelliği ve tohum bankası konumunun da dikkate alınması yaprak karakter değişkenliğini daha iyi açıklamıştır. Büyüme şeklinde üç yaprak karakterinde tutarlı bir örüntü gözlemlenmemesine rağmen, çalılar ve büyük çalılar arasında SLA ve yaprak kalınlığında farklar olduğuna ve bodur çalıların diğer büyüme şekillerine göre daha küçük yapraklara sahip olduğuna dair kanıtlar bulduk. Vejetasyon tipi de yaprak karakter çeşitliliğinin önemli bir kısmını açıklamıştır. Özellikle, kapalı habitatlardaki bitki komüniteleri açık olanlardan daha büyük yaprak alanına sahipken, bodur çalılıklardaki komüniteler diğer vejetasyon tiplerine göre daha yüksek SLA, daha düşük yaprak kalınlığı ve daha düşük yaprak alanına sahipti. Yeniden sürgün verenler ve veremeyenler arasında SLA farklılıklarını açıklayan önemli bir filogenetik etki dışında, iklim ve filogeni elde edilen sonuçlara sınırlı miktarda katkıda bulunmuştur. Sonuçlarımız, Akdeniz bitki komünitelerindeki bitki karakter değişkenliğinin şekillenmesinde aralarında büyüme şekli, rejenerasyon modu ve vejetasyon tipinin de yer aldığı çok sayıda sürücü etkenin sorumlu olduğunu önermektedir.

## 1 INTRODUCTION

Plant functional traits are morphological, physiological, and phenological characteristics of plant species affecting their growth, reproduction, survival, and response to changing environments (Garnier et al., 2004, 2016; Kühn et al., 2021; Pérez‐Harguindeguy et al., 2013; Violle et al., 2007). Among these, leaf functional traits are integral to understanding ecological strategies (Westoby, 1998) and determine how a species adapts to and interacts with environmental challenges (Dong et al., 2020). They serve as indicators of ecosystem functioning in community‐level assessments (de Bello et al., 2010; Díaz et al., 2004; Stanisci et al., 2020) and include a range of characteristics that influence a plant's performance and its interactions within a community (Reich et al., 2003). Recent studies emphasize the universal importance of leaf functional traits in deciphering ecological patterns across diverse ecosystems, shedding light on their role as integrative measures of plant performance and resource‐use strategies (Laughlin et al., 2020; Siefert et al., 2015). Additionally, variations in these traits at both the species and community levels can provide critical information about a plant's competitive ability, resilience, and overall contribution to ecosystem processes (Albert et al., 2010; Díaz et al., 2016).

Although many leaf traits are phylogenetically conserved (Homeier et al., 2021), among‐ and within‐species variability also contributes a significant amount to the geographic variation of many leaf traits (Dong et al., 2020; Messier et al., 2017), even at the community level (Siefert et al., 2015). Drivers of leaf trait variability differ across various biomes globally, mainly due to their difference in environmental and disturbance processes (Llerena‐Zambrano et al., 2021). Climate is among the main drivers of variability in some leaf traits, including leaf area (Dong et al., 2020; Geekiyanage et al., 2017; Llerena‐Zambrano et al., 2021), SLA (Homeier et al., 2021; Shi et al., 2018; Wang et al., 2015), and leaf thickness (Geekiyanage et al., 2017; Homeier et al., 2021; Shi et al., 2018). Therefore, studying leaf trait variability at the community level is crucial to understanding the potential of local communities to adapt to new environmental conditions such as prolonged and/or intensified droughts associated with climate change. Leaf trait variation among species and communities were studied in several aspects, such as along elevational gradients (e.g., Homeier et al., 2021; Llerena‐Zambrano et al., 2021) and in comparing different environmental conditions (e.g., Geekiyanage et al., 2017; Markesteijn et al., 2007; Wang et al., 2015), functional groups (Jin et al., 2014), and vegetation types (Shi et al., 2018). However, as environmental conditions substantially differ at regional or local scales, more studies are still needed for a comprehensive understanding of leaf trait variability across various scales in different terrestrial biomes.

Mediterranean plant species have developed strategies to cope with limiting resources, especially in relation to drought conditions (Altieri et al., 2015; Hernández et al., 2010; Maseyk et al., 2011; Mereu et al., 2009). In Mediterranean plants, leaves have evolved to reduce water loss (Chirino et al., 2017; Hernández et al., 2010; Valencia et al., 2016). Dominant woody species in Mediterranean vegetation, characterized by small and sclerophyllous leaves with thick epidermal walls and cuticle (Ackerly et al., 2002; Paula & Pausas, 2006), have adapted to low water availability (Gillison, 2019; Paula & Pausas, 2006) and have developed conservative water use strategy (Kühn et al., 2021). Moreover, drier environments in the Mediterranean Basin host more resource‐conservative species than wetter ones (Garnier et al., 2019). Leaf trait variability in Mediterranean plant communities, however, can be influenced by factors beyond climate, including among‐species differences (Hulshof & Swenson, 2010), local environmental conditions like light, humidity, or soil nutrients (Campetella et al., 2019; Domínguez et al., 2012), and resprouting ability (Paula & Pausas, 2006).

Mediterranean Basin ecosystems have long experienced several types of natural disturbances, including drought, herbivory, and fire (Lavorel, 1999). Since the onset of the Holocene, human‐driven disturbances such as agricultural activities, domestic herbivory, and other kinds of land uses have also significantly contributed to shaping the Mediterranean Basin landscapes (Naveh & Carmel, 2004). Therefore, both natural and human‐caused disturbances are considered important drivers of the current occurrence and distribution of various vegetation types in the Mediterranean Basin. Forests, shrublands, and scrublands are three major vegetation types in the Mediterranean Basin (Arianoutsou, 1998; Blondel & Aronson, 1999; Kavgacı et al., 2017; Keeley et al., 2012), and they considerably vary in form, structure, diversity, and human use (Keeley et al., 2012). Moreover, functionally significant differences between open and closed states of forests in several terrestrial biomes on Earth are also well‐known (Bond, 2019; Pausas & Bond, 2020). Given that similar disparities may exist across various vegetation types in the Mediterranean Basin, such as open or closed forests and shrublands (Tüfekcioğlu & Tavşanoğlu, 2022), examining the functional differences between the open and closed states of these major vegetation types can more comprehensively represent the diverse species, functional groups, and plant communities in the region. Functional group classifications also improve our understanding of ecosystem function and processes (Díaz Barradas et al., 2009) and allow us to follow the patterns of vegetation recovery after disturbances over a long‐term period (Kazanis & Arianoutsou, 2004; Tavşanoğlu & Gürkan, 2014). Although various Mediterranean vegetation types can be quite complex regarding growth forms and regeneration modes (Tüfekcioğlu & Tavşanoğlu, 2022), different trends can be observed during post‐disturbance recovery in different vegetation communities. The resprouting ability plays an essential role in vegetation recovery in the Mediterranean Basin after particular disturbances, such as wildfire and drought (Pausas et al., 2016). Moreover, resprouting after disturbance is an important plant trait associated with other plant traits, including several leaf traits (Hernández et al., 2011; Paula & Pausas, 2006). Mediterranean plant functional classification is more intricately defined by regeneration strategy, encompassing both resprouting and seedling establishment abilities (Pausas et al., 2004). This approach broadens the basic dichotomy of resprouters and non‐resprouters by incorporating the efficacy of seedling establishment from propagules, distinguishing between propagule persisters and non‐persisters (Saura‐Mas & Lloret, 2007; Tavşanoğlu & Gürkan, 2014). This distinction is also important to include seed germination and seedling survival, which is a critical trait under the summer‐dry conditions of the Mediterranean climate. Given that fire has been a key evolutionary force shaping various plant traits (Keeley et al., 2012), these classification approaches offer vital insights into the dynamics of plant communities in Mediterranean ecosystems.

In this study, we aimed to elucidate the drivers shaping leaf trait variability in Mediterranean woody plant communities. Specifically, we ask the question whether leaf trait variability at species and community levels in Mediterranean woody vegetation is explained by growth form, regeneration mode, and vegetation type. We expected to find that functional groups account for a significant part of leaf trait variability at the species level, and vegetation type is another driver responsible for creating leaf trait variability at the community level. To test these hypotheses, we measured leaf traits of several species in woody plant communities across various Mediterranean vegetation types (semi‐closed forest, open forest, closed shrubland, open shrubland, and scrubland). Then, we examined the variability in leaf traits among species and communities based on functional groups (the growth form and regeneration mode) and vegetation type.

## 2 MATERIALS AND METHODS

### 2.1 Study area and sites

The study area was in southwestern Anatolia (Türkiye), which is under a Mediterranean climate characterized by cool, wet winters and hot, dry summers and dominated by Mediterranean vegetation types, including several open and closed vegetation states (Tüfekcioğlu & Tavşanoğlu, 2022). We established our study sites in the five most frequent vegetation types of the region: semi‐closed Turkish pine (*Pinus brutia*) forest with total pine cover between 11% and 40% (hereafter: semi‐closed forest), open Turkish pine forest with total pine cover <10% (hereafter: open forest), closed maquis shrubland with total canopy cover between 11% and 100% (hereafter: closed shrubland), open maquis shrubland with total canopy cover <10% (hereafter: open shrubland), and phrygana scrubland dominated by *Sarcopoterium spinosum* (hereafter: scrubland). The selected study sites were unmanaged and had not been burned for several years, and they were in their mature successional stages. Although these vegetation types share several plant species, there were also significant differences in their woody species composition (Tüfekcioğlu & Tavşanoğlu, 2022). Specifically, *Phillyrea latifolia* and *Quercus coccifera* were accompanying to Turkish pine in closed and open forests, while *Arbutus andrachne*, *Olea europea*, and *Q*. *coccifera* were dominant in closed shrublands and *Q*. *coccifera*, *Cistus salviifolius*, and *Genista acanthoclada* in open shrublands. We selected 28 study sites, 1 ha in size, distributed them to different vegetation types by considering their relative area covered in the study area (six for the semi‐closed forest, eight for the open forest, four for the closed shrubland, six for the open shrubland, and four for the scrubland). A further detailed explanation of the study sites is given by Tüfekcioğlu and Tavşanoğlu (2022).

### 2.2 Leaf sampling and transect surveys

We collected leaf samples within study sites to measure leaf traits of individual plants in the dry period of the region, i.e., between May and September 2019, and only in September 2020 due to COVID‐19 mobility restrictions. Following Pérez‐Harguindeguy et al. (2013), we sampled 10 leaves from mature and healthy‐looking individuals located in unshaded locations. In order to avoid dehydration after collection, we wrapped leaf samples in moist paper and put them in sealed plastic bags just after we collected the leaves. We blew into the bags before closing them to allow more carbon dioxide inside to minimize the water loss due to transpiration. Finally, bags including leaf samples were stored in a cool box in the field until they were put in a refrigerator at 4°C. We made further processing of leaf samples within 24 h after the collection.

In total, we collected leaf samples from 857 individuals of 38 species, of which 709 individuals of 37 species in 2019 and 148 individuals of 23 species in 2020. Since all plant species were not found in all study sites, the number of individuals sampled varied among study sites and vegetation types. Therefore, we obtained more leaf samples from the most frequent species in comparison to rarer ones (Table S1).

In each study site, we established three belt transects 10 × 40 m and 10 × 30 m in size (according to topography) except for a study site including two transects due to topographic limitations. In total, we sampled 83 belt transects. We counted all mature individuals of woody species in each belt transect to obtain abundance data for further use in community weighted mean analyses. Some of those individuals were the same ones on which we made leaf sampling.

### 2.3 Trait measurements

We studied leaf area, leaf thickness, and specific leaf area (SLA), which are leaf traits informing resource use and ecosystem properties (Li et al., 2022; Paula & Pausas, 2006; Roche et al., 2004). SLA is often cited as a key plant functional trait (Garnier et al., 2004; Reich et al., 1992; Roche et al., 2004; Wilson et al., 1999), leaf area dramatically affects the energy acquired by a leaf and is related to the water balance (Díaz et al., 2016), and leaf thickness acts as a proxy for a leaf's physical strength (Pérez‐Harguindeguy et al., 2013).

We measured all three leaf traits for each sampled individual. We followed the Pérez‐Harguindeguy et al. (2013) protocols for measuring leaf traits. We made one measurement on the lamina of each leaf by using a digital micrometer to measure leaf thickness. To measure the leaf area, we scanned the leaves collected from the field and then calculated their one‐sided area using the ImageJ program (Rasband, 2012). To obtain SLA values for each individual plant, we first weighted leaves using a digital scale after they were dried in the oven at 70°C for 72 h to determine the oven‐dry mass of leaves (Pérez‐Harguindeguy et al., 2013). After this process, SLA values were calculated by dividing the average leaf area of each individual by the total oven‐dry mass value.

### 2.4 Functional groups

We used three functional grouping systems in this study. First, we classified woody species according to their growth forms as subshrub, shrub, large shrub, tree, and liana. This classification is based on Tavşanoğlu and Pausas (2018) and field observations. Second, we used post‐fire resprouting ability as a binary classification system for regeneration mode: resprouters and non‐resprouters. Finally, we assign species into one of the four regeneration strategy classes reflecting the regeneration properties of species in more detail: (1) non‐resprouter propagule persisters with a canopy seed bank (R − P + c), non‐resprouter propagule persisters with a soil seed bank (R − P + s), resprouter propagule‐non‐persisters (R + P−), and resprouter propagule‐persisters with a soil seed bank (R + P). This regeneration mode classification is based on Pausas et al. (2004) with addition of seed bank location for propagule persisters (Pausas, 1999; Tavşanoğlu & Gürkan, 2014). Resprouting ability and propagule persistence traits used in the latter two classification systems are based on Tavşanoğlu and Pausas (2018) and field observations. Since data for resprouting ability and propagule persistence for some species are missing in the literature and BROT database (Tavşanoğlu & Pausas, 2018), and the resprouting ability varies at the population level in some species, we excluded some species from resprouting ability and regeneration strategy analyses. Consequently, although we used data from all 38 species and 857 individuals for growth form analysis, we included 31 species and 784 individuals in resprouting ability analysis and 30 species and 732 individuals in regeneration strategy analysis (Table S1).

### 2.5 Data analysis

First, we revealed among‐species variation in the studied leaf traits (i.e., SLA, leaf thickness, and leaf area). We only used species with leaf samples from at least five individuals; thus, the data consisted of 837 individuals belonging to 31 species in this analysis. We made ridgeline plots to visualize within‐ and among‐species variability for each trait. For further investigation of trait variability, we assessed the range (min. and max.) of trait values for each species. Then, we performed a general linear model to reveal differences among species. Moreover, as a proxy for among‐species variability in each leaf trait, we calculated the coefficient of variation for each trait based on mean trait data at the species level.

We compared functional group classes for leaf thickness, leaf area, and SLA for each functional grouping system. To visualize and analyze the differences among functional group classes, we made violin plots to show the interquartile range and the lower/upper adjacent values and performed linear mixed effects models (LME) for each trait for functional groups. In these models, functional group class and species were considered fixed and random factors, respectively. Since the number of sampled individuals varied for each functional group class, we performed the LME analysis by weighting data with the number of sampled individuals. We calculated the mean and standard error of leaf traits for each functional group and made multiple comparisons following LME analyses by estimating marginal means based on Tukey HSD adjustment method for different functional group classes. Trait data were log‐transformed before LME analyses to improve the linearity of relationships, and model residuals were visually checked for homoscedasticity.

For the community analysis, we calculated community weighted mean (CWM) by weighting leaf trait values with the abundance of species for each transect (i.e., plant community) (Garnier et al., 2004). In this analysis, we used only trait data of individuals with abundance data sampled within belt transects. For CWM calculation, we used average values for leaf traits for each species. Missing trait data for the species that we could not collect leaf samples in the field (from summer‐deciduous species as the time of the leaf fall coincides with the sampling period and from individuals full of unhealthy leaves) were obtained from the BROT database (Tavşanoğlu & Pausas, 2018), Hacettepe University Functional Ecology Lab. data (Aktepe, 2021; Coşgun, 2022), and the relevant literature (Elmas & Kutbay, 2015; Liakoura et al., 2001; Merchant, 1998; Specht, 1988). Even though there were still some species whose trait data are missing (6, 14, and 8 species for SLA, leaf thickness, and leaf area, respectively), we concluded that this missing data would not cause any problem for the community analyses since many of these species had low abundance in the field.

Following CWM analysis, we performed LME for each trait for vegetation types. In these models, the vegetation type and transect were considered as the fixed and random factors, respectively. We calculated the mean and standard error of leaf traits for each vegetation type and made multiple comparisons following LME analyses by estimating marginal means based on Tukey HSD adjustment method for different vegetation types. Trait data were log‐transformed before LME analyses to improve the linearity of relationships, and model residuals were visually checked for homoscedasticity.

We also implemented a principal components analysis (PCA) incorporating three leaf traits considered in the study. This analysis was used to highlight the differences in leaf traits across various vegetation types and functional group classes, thereby facilitating a deeper examination into how these traits vary among the different vegetation categories and functional groups.

To test how traits vary across climatic gradients, we performed a linear regression analysis to examine the relationships between the studied leaf traits and temperature and precipitation variables (specifically, annual mean temperature, annual total precipitation, and precipitation of the driest quarter). Climate data were obtained from the WorldClim database (WorldClim, 2023). We also evaluated bedrock type as a potential explanatory environmental variable for leaf trait variability, testing the data using PCA based on four bedrock type categories: alluvial, clastic sedimentary rock, limestone, and volcanic. Besides that, to explore the varitation in community traits, we calculated the most frequently‐used distance‐based functional diversity indices (Garnier et al., 2016) as functional richness (FRic), functional evenness (FEve), functional divergence (FDiv), and functional dispersion (FDis) for each vegetation type to compare leaf functional traits among vegetation types at the community level. In this analysis, we used mean trait values for each species and calculated these indices based on the community compositions of five vegetation type categories.

Ultimately, to elucidate the effect of phylogeny on the model results, we also tested our hypothesis by taking into account the phylogenetic relatedness of species with respect to leaf traits. To do this, we performed phylogenetic linear models and compared the results with non‐phylogenetic models. To assembe the phylogenetic tree used in these models, we used ‘GBOTB.extended’ mega‐tree for vascular plants implemented in *V.PhyloMaker* package in R (Jin & Qian, 2019).

All the analyses were performed in the R environment (R Core Team, 2020). We used *ggridges* package (Wilke, 2021) for drawing ridgeline plots, *ggplot2* package (Wickham, 2016) for drawing violin and box plots, *lme* function in the *nlme* package (Pinheiro & Bates, 2000) for performing LMEs, *pairs* and *emmeans* functions in the *emmeans* package (Lenth, 2020) for estimating marginal means following LMEs, *prcomp* and *pairwise*. *adonis* functions in the *vegan* package (Oksanen et al., 2019) for performing PCA, *dbFD* function in the *FD* package (Laliberté et al., 2023) for calculating distance‐based functional diversity indices, *phylo.maker* function in the *V.PhyloMaker* package (Jin & Qian, 2019) for constructing phylogenetic tree and *phylolm* function in the *phylolm* package (Ho et al., 2022) to run phylogenetic models.

## 3 RESULTS

### 3.1 Variation among species

**FIGURE 1:**
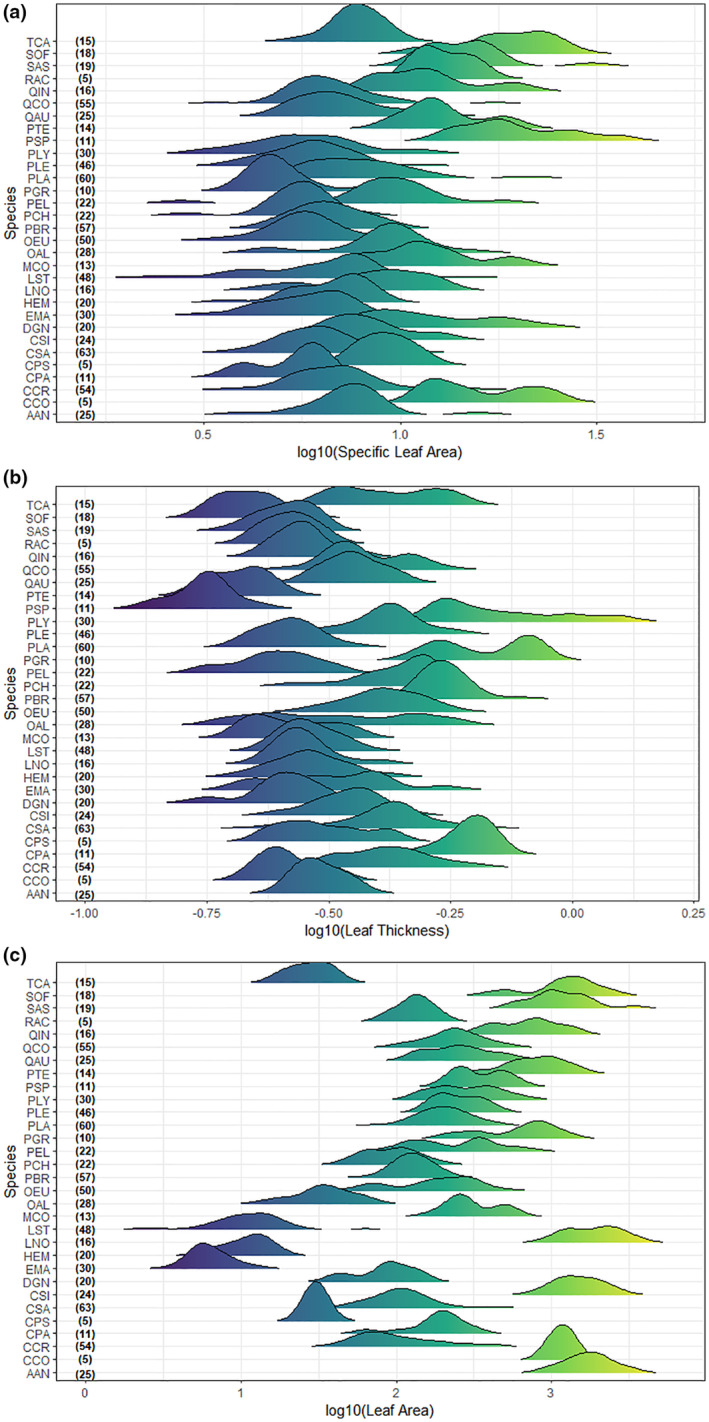
Within‐ and among‐species variation in the specific leaf area (a; mm^2^ mg^−1^), leaf thickness (b; mm), and leaf area (c; mm^2^). Each graph within the figures indicates within‐species variability for the corresponding species. Species specified by codes are as follows: AAN, *Arbutus andrachne*; AUN, *Arbutus unedo*; CVI, *Calicotome villosa*; CSI, *Ceratonia siliqua*; CCR, *Cistus creticus*; CPA, *Cistus parviflorus*; CSA, *Cistus salviifolius*; CCO, *Cotinus coggygria*; CSE, *Cupressus sempervirens*; CPS, *Cytisopsis pseudocytisus*; DGN, *Daphne gnidioides*; DSE, *Daphne sericea*; EMA, *Erica manipuliflora*; HEM, *Hypericum empetrifolium*; JOX, *Juniperus oxycedrus*; LNO, *Laurus nobilis*; LST, *Lavandula stoechas*; MCO, *Myrtus communis*; OEU, *Olea europea*; OAL, *Osyris alba*; PSP, *Paliurus spina‐christi*; PLA, *Phillyrea latifolia*; PGR, *Phlomis grandiflora*; PLY, *Phlomis lycia*; PBR, *Pinus brutia*; PLE, *Pistacia lentiscus*; PTE, *Pistacia terebinthus*; PCH, *Ptilostemon chamaepeuce*; PEL, *Pyrus elaeagnifolia*; QAU, *Quercus aucheri*; QCO, *Quercus coccifera*; QIN, *Quercus infectoria*; QIT, *Quercus ithaburensis*; RPU, *Rhamnus punctata*; RAC, *Ruscus aculeatus*; SAS, *Smilax aspera*; SOF, *Styrax officinalis*; TCA, *Thymbra capitata*. The numbers in parentheses are the sample sizes.

Leaf traits showed substantial variation both within‐ and among‐species (Figure 1, Table S2). Leaf area had the highest coefficient of variation value (116.2) in comparison to other traits (44.8 and 39.1 for SLA and leaf thickness, respectively); therefore, it was the most variable trait among species. Differences among species were significant in all leaf traits (for SLA *F* = 38.4, leaf thickness *F* = 67.9, and leaf area *F* = 279.8, *p* < .0001 for all). The variability among species in SLA and leaf area in our study falls into the range for the existing trait measurements in the Mediterranean Basin: 1.55 and 32.36 mm^2^ mg^−1^ for SLA, and 3.0 and 2610.0 mm^2^ for leaf area (except two extreme values up to 11,968.0 mm^2^) (in comparison to the ranges in the BROT database; Tavşanoğlu & Pausas, 2018).

### 3.2 Functional groups

**FIGURE 2:**
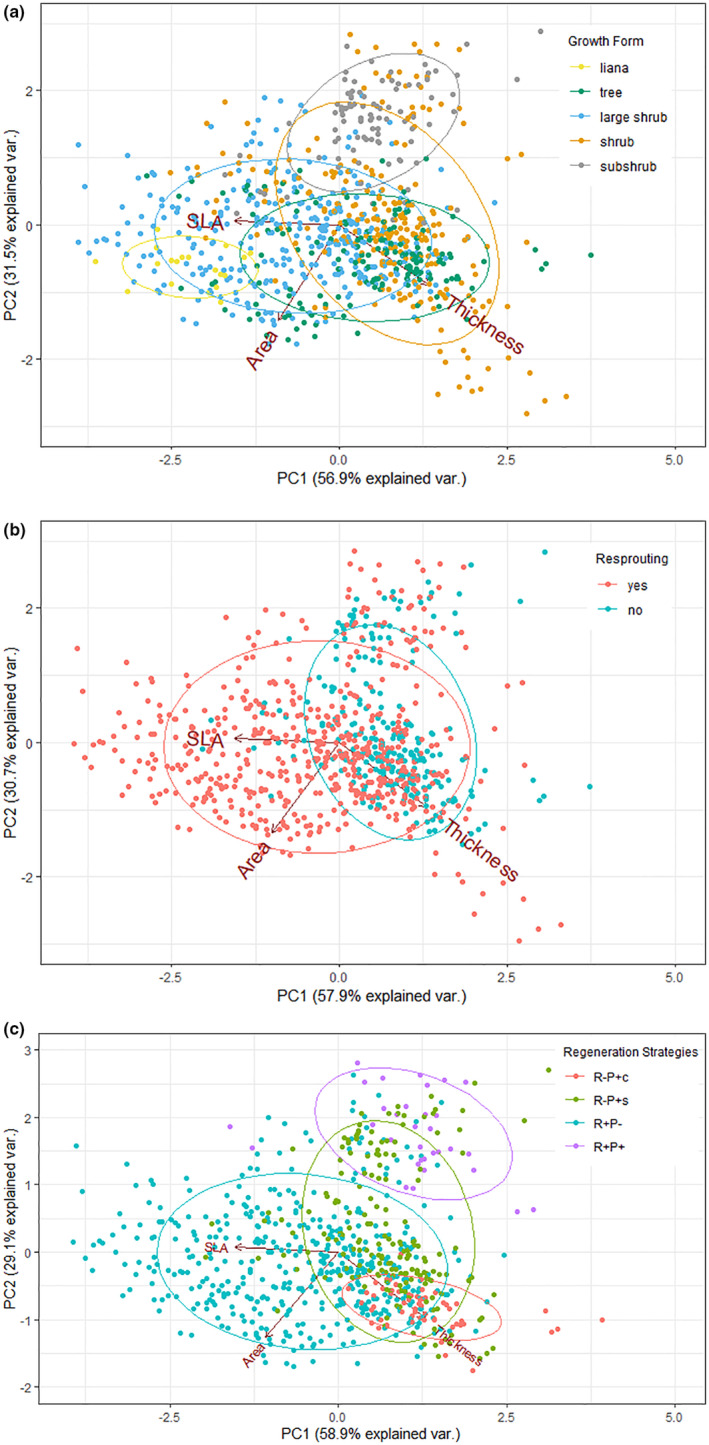
Principal component analysis graph for specific leaf area, leaf thickness, and leaf area for different functional group classifications according to (a) growth form, (b) resprouting ability, and (c) regeneration strategy (R − P + c: non‐resprouter propagule persisters with a canopy seed bank; R − P + s: non‐resprouter propagule persisters with a soil seed bank; R + P−: resprouter propagule‐non‐persisters; R + P+: resprouter propagule‐persisters with a soil seed bank). Different colors represent different functional groups. Each data point is the mean value in the study area of individuals measured, and eclipses indicate the standard deviation of each group.

Functional groups accounted for a significant part of the variability in the studied leaf traits. Different functional grouping systems explained trait variability at various degrees (Figure 2, Table S3). Accordingly, we found evidence for differences among functional groups in different classification systems. Specifically, the growth form and regeneration strategy accounted for ca. 30% of the variability in three leaf traits (PCA analysis, *R* ^2^ = .28 and *R* ^2^ = .26, respectively, both *p* = .001), while the resprouting ability explained only ca. 10% of the total variability in leaf traits (*R* ^2^ = .10, *p* = .001).

**FIGURE 3:**
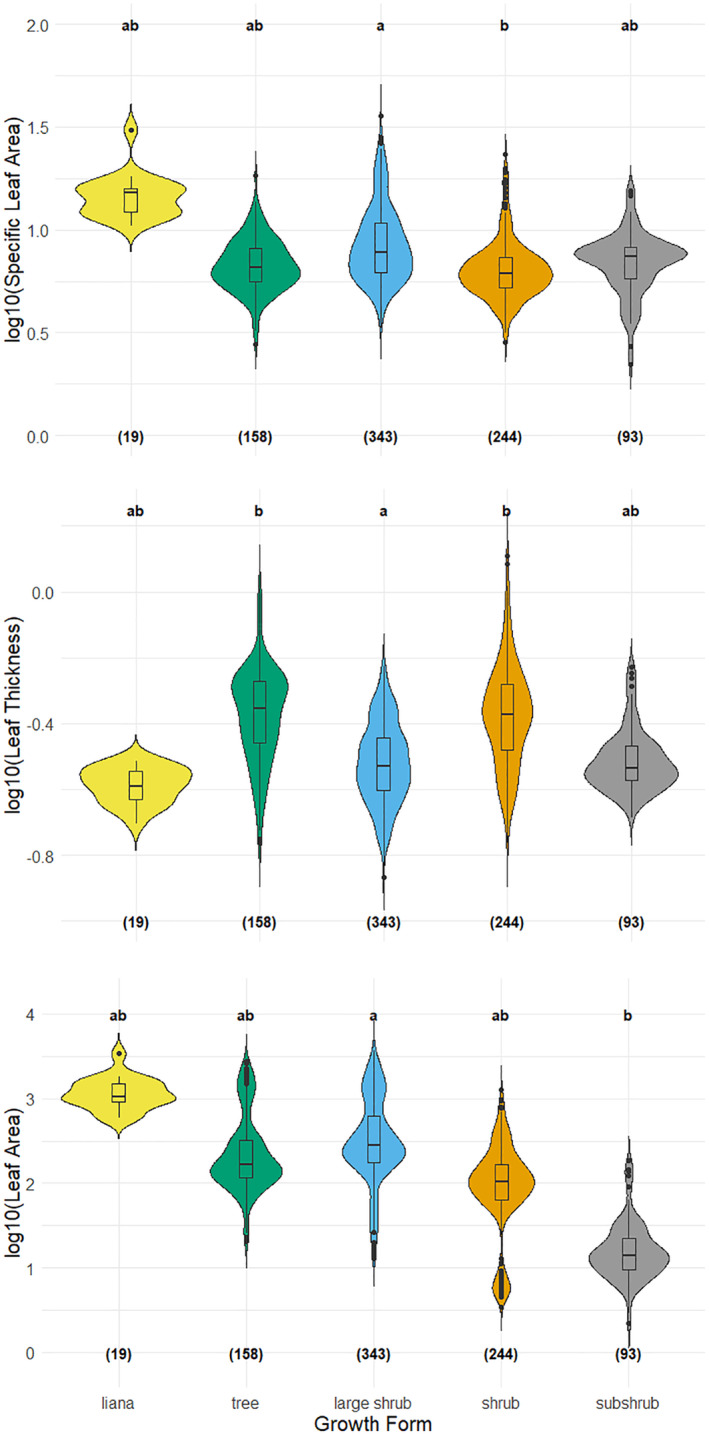
Comparison of specific leaf area, leaf thickness, and leaf area for different growth forms. The letters above indicate statistical test results as having different letters mean a significant difference between two groups, and the numbers in parentheses at the bottom represent the sample size (i.e., individuals measured).

We observed the lowest SLA values in individuals of some shrubs (*Erica manipuliflora*, *Phlomis lycia*, and *Ptilostemon chamaepeuce*) and the highest ones in those of some large shrubs (*Cotinus coggygria*, *Paliurus spina‐christi*, and *Styrax officinalis*), while other growth forms exhibited no clear pattern (Table S2). Indeed, there was evidence for the difference in SLA between shrubs and large shrubs (Figure 3, Table S4). We also observed a difference in leaf thickness values between shrubs and large shrubs (higher and lower values, respectively), but trees also had higher leaf thickness than large shrubs (Figure 3, Table S4). Subshrubs had smaller leaves than any other growth form group (Figure 3, Table S4), and the species with the largest leaves were all large shrubs (*Arbutus andrachne*, *Ceratonia silique*, *Cotinus coggygria*, and *Laurus nobilis*, Table S2).

**FIGURE 4:**
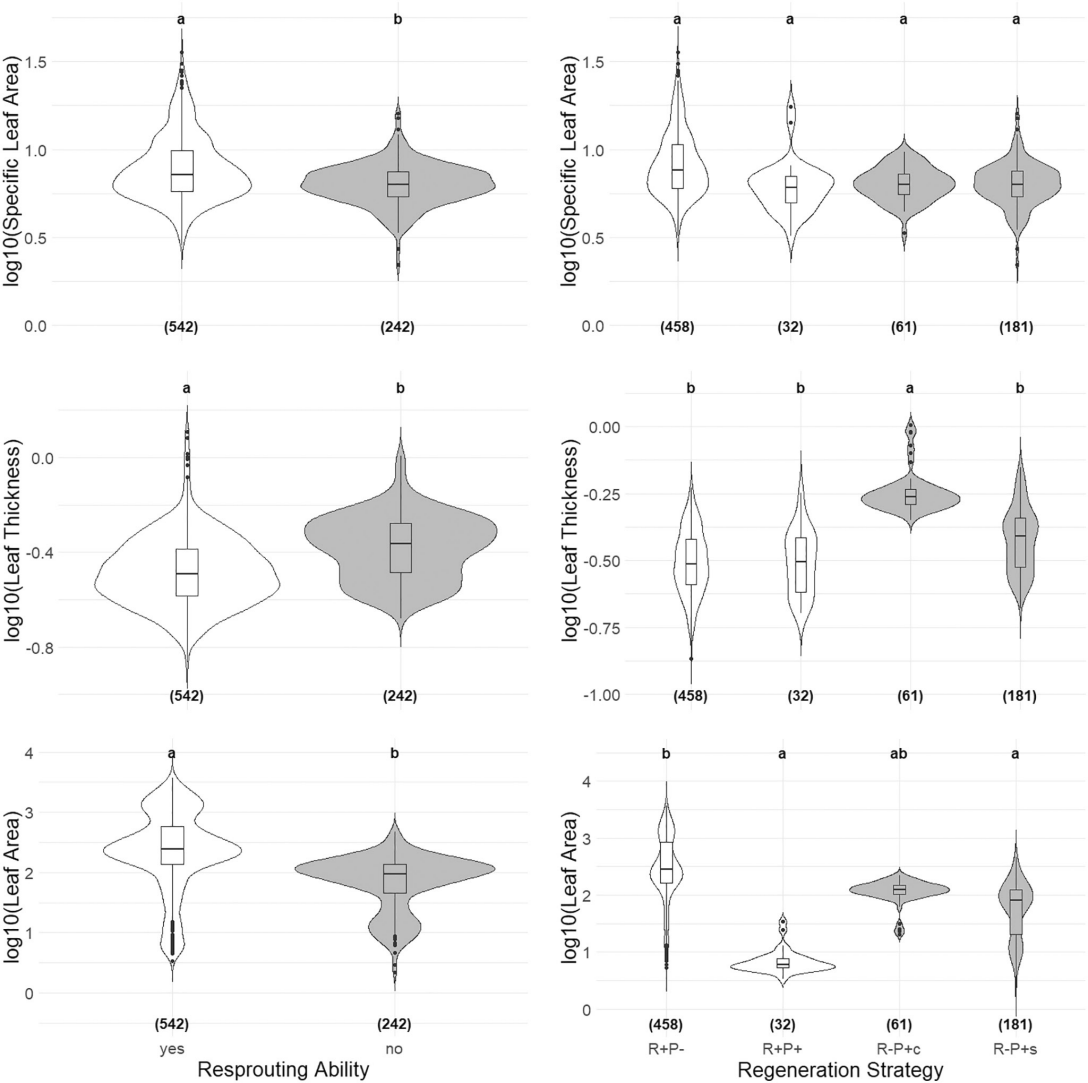
Comparison of specific leaf area, leaf thickness, and leaf area for resprouting (yes/no, at the left panel) and regeneration strategies classes (at the right panel). The letters above indicate statistical test results as having different letters mean a significant difference between two groups, and the numbers in parentheses at the bottom represent the sample size (i.e., individuals measured). In all graphs, white and gray plots represent resprouters and non‐resprouters, respectively.

We obtained consistent results using two alternative grouping approaches based on the regeneration mode; resprouting ability and regeneration strategy (Figure 4, Table S4). In general, resprouters had higher SLA and leaf area and lower leaf thickness values than non‐resprouters at both species (Table S2) and functional group levels (Figure 4, Table S4). Although we found no evidence of difference in SLA among regeneration strategy groups, a clear distinction was obtained when comparing resprouting ability classes (resprouters vs. non‐resprouters) (Figure 4). Moreover, this difference can be attributed to the higher SLA values of species with R + P− strategy but not to those with the R + P+ strategy with similar SLA values with non‐resprouters (Figure 4, Table S4). In terms of leaf thickness, the leaves of the non‐resprouters were thicker than the resprouters, and this difference was mainly due to *Pinus brutia* with the R − P + c strategy. Similar to the pattern we observed in SLA, we found evidence that resprouters had larger leaves than non‐resprouters, but this difference was due solely to R + P− strategists but not species with R + P+ strategy (Figure 4, Table S4).

### 3.3 Vegetation type

**FIGURE 5:**
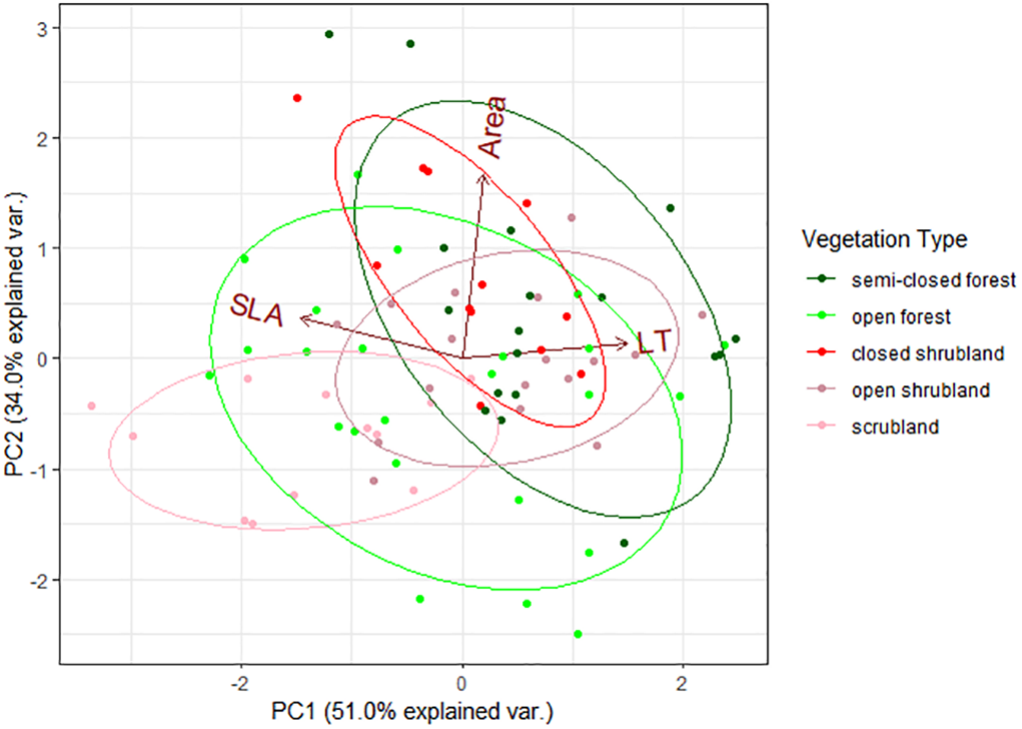
Principal component analysis graph of community weighted mean values for specific leaf area, leaf thickness, and leaf area among different vegetation types. Each data point is community‐weighted mean value of each transect, and eclipses indicate the standard deviation of each vegetation type.

**FIGURE 6:**
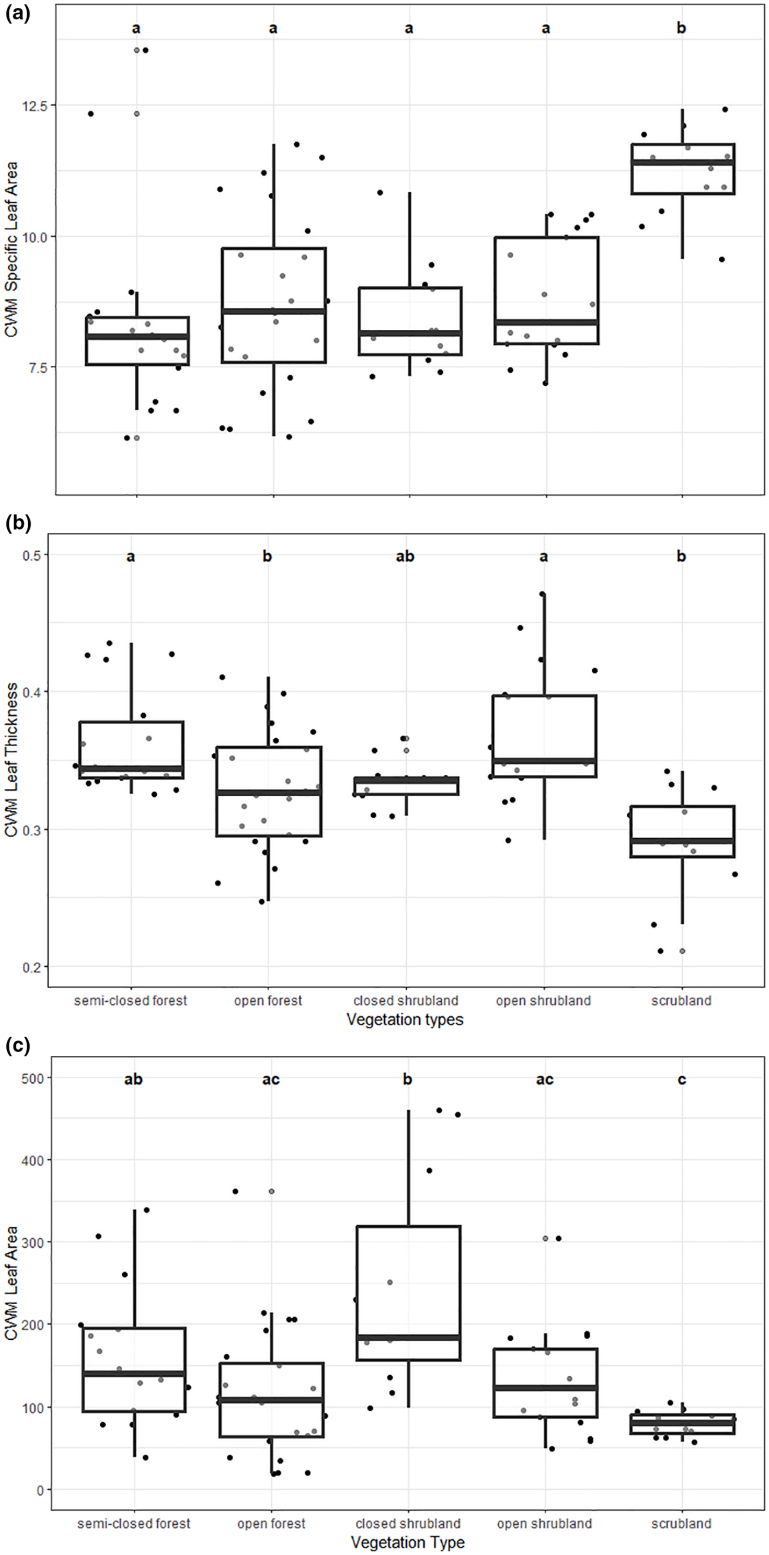
Comparison of community weighted mean values for specific leaf area (a), leaf thickness (b), and leaf area (c) among vegetation types. Each data point is community‐weighted mean value of each transect. The letters above indicate statistical test results as having different letters mean a significant difference between two groups.

Since the abundance of species varied in plant communities belonging to different vegetation types, the contribution of each species to the community trait mean also differed among vegetation types (Table S5). Consequently, PCA analysis showed that the vegetation type explains a considerable variation in leaf traits among local plant communities (*R* ^2^ = .29, *p* = .001, Figure 5). Plant communities in semi‐closed forest, open forest, closed shrubland, and open shrubland were relatively similar for the studied leaf traits, while scrubland differed from these vegetation types (Figure 5, Table S6). Scrubland had higher SLA, lower leaf thickness, and lower leaf area than other vegetation types in many cases (Figure 6, Table S7). Although this general trend, we provided no evidence for the difference between scrubland and open forest regarding leaf thickness and leaf area (Figure 6, Table S7). Other exceptions we observed were including the similar leaf area values obtained for scrubland and open shrubland, the lower leaf thickness in the open forest than in semi‐closed forest and open shrubland, and the higher leaf area in closed shrubland than in open forest and open shrubland (Figure 6, Table S7). We revealed that a few species dominated the scrublands with relatively higher SLA, lower leaf thickness, or lower leaf area values (*Cistus creticus*, *Genista acanthoclada*, *Sarcopoterium spinosum*, and *Thymbra capitata*, Table S5) were responsible for the difference in leaf traits between the communities in scrubland and those of other vegetation types.

Calculated functional diversity indices showed considerable differences across vegetation types. The minimum functional richness was observed in scrublands, showcasing a distinct disparity among the vegetation types (Table S8). Conversely, the functional evenness values reached their peak in semi‐closed forest and scrubland vegetations, while the maximum functional divergence and dispersion values were evident in closed habitats, namely semi‐closed forest and closed shrubland (Table S8). Notably, scrublands exhibited a lower value in comparison to other vegetation types with respect to the functional dispersion index (Table S8).

### 3.4 Climate, bedrock type, and phylogeny

Among the climatic variables tested, only leaf area showed a weak association with annual total precipitation (*R* ^2^ = .104, *p* = .052, Figure S1) and precipitation of the driest quarter (*R* ^2^ = .153, *p* = .022, Figure S1). None of other combinations among climatic variables and traits considered in the study demonstrated significant associations (*R* ^2^ ≤ .06, *p* > .05). The variability in leaf traits appeared to be independent of the bedrock type, as PCA did not differentiate any of the bedrock type categories (Figure S2). Additionally, phylogeny had also limited effect on the results as non‐phylogenetic models exhibited superior explanatory power for all leaf traits studied, except for the association between the resprouting ability and SLA (Table S9).

## 4 DISCUSSION

Our results reveal the significant within‐ and among‐species variability in leaf traits in Mediterranean woody plants. Despite this substantial variability, our study provides evidence that leaf trait variation in Mediterranean woody plants can be partly explained by plant functional groups, including growth form, resprouting ability, and regeneration strategy. Our analyses further showed that vegetation type also accounts for a significant proportion of leaf trait variability at the local community level. Therefore, the results supported our hypotheses and initial expectations.

The observed variability in leaf traits among Mediterranean woody species in our study underscores the complex interactions between species‐specific adaptations and environmental pressures in Mediterranean ecosystems (de la Riva et al., 2018). Our results align with global patterns of leaf trait variability, where species‐level differences often reflect distinct ecological strategies and environmental adaptations (Reich, 2014; Wright et al., 2004). Specifically, the substantial variation in SLA and leaf area among species in the studied region is consistent with the observed variability at the Mediterranean Basin and global scales (Díaz et al., 2016; Tavşanoğlu & Pausas, 2018). This suggests a broad ecological strategy spectrum in Mediterranean woody species even at the regional level. This extensive interspecific trait variation can be attributed to the heterogeneity of Mediterranean landscapes, as demonstrated in our study, and the varying degrees of water stress experienced by these plant communities.

The distribution of plant trait values among different vegetation types has drawn little research attention globally, but such studies provide notable insights into our understanding of the evolution and ecology of biomes and regional floras (Dantas & Pausas, 2020). In the Mediterranean Basin, differences in SLA values have been observed across various vegetation types along with aridity or elevational gradients (de la Riva et al., 2018; Navarro & Hidalgo‐Triana, 2021) and between early and late successional stages (Garnier et al., 2004; Kazakou et al., 2006). In our study area, scrublands differed considerably from the other vegetation types, as we found the highest SLA and the lowest leaf thickness and leaf area in these plant communities. Although this difference was due to trait values of a few species that dominated scrublands, it has an ecological significance regarding the response of the plant community to environmental conditions. Scrublands also exhibited the lowest functional richness and functional dispersion, yet they showed functional evenness and divergence values comparable to other vegetation types. This pattern may imply a more specialized adaptation to the scrubland environment. At the same time, despite the low species richness, the existing species in scrublands appear to be well‐distributed across the available functional niche space. This suggests that scrublands form a balanced, yet specialized, ecosystem type.

Leaf area also showed a pattern among vegetation types, such that the plant community in closed vegetations (i.e., closed forest and closed shrubland) had larger community‐weighted mean leaf size than open ones (open forest, open shrubland, and scrubland). Species with lower SLA, smaller leaves, and higher leaf thickness are well known to be more tolerant to drought conditions in many ecosystems (Ackerly et al., 2002; Costa‐Saura et al., 2016; de la Riva et al., 2018; Kühn et al., 2021; Nunes et al., 2017; Wright et al., 2017). Our results on SLA and leaf thickness might hint at differential adaptations, potentially indicating more efficient water usage and enhanced photosynthetic efficiency per unit leaf area in Mediterranean forests and shrublands (regardless of their openness status), while scrublands could have more capacity for drought resistance. However, it is important to note that our study did not directly measure water use efficiency, photosynthetic capacity, or drought resistance. Considering the prolonged drought conditions in the Mediterranean Basin (and specifically in our study area), plant species in scrublands may have to compensate for their high SLA and low leaf thickness with their small leaf area to have some drought resistance. Moreover, since SLA has a positive relationship with the relative growth rate (Violle et al., 2007), the results also suggest that plant communities in scrublands consist of species with faster growth rates than other vegetation types. Thus, our results may indicate that scrublands are at different place of the slow‐fast continuum of the life history than forests and shrublands at the community level in the Mediterranean Basin. Therefore, the plant community in scrublands can be expected to have better performance under frequent disturbances (such as fire and herbivory) but worse in the case of drought than shrublands and forests, and vice versa.

Due to the prolonged summer drought, specific leaf traits are expected to be filtered by regional climatic conditions in the Mediterranean Basin. This filtering process may have resulted in assembling plant communities exhibiting adaptations to drought conditions. For instance, sclerophyll leaves are characteristic of woody plant species in Mediterranean‐type ecosystems worldwide; even their floras share no or ancient evolutionary origins (Mooney & Dunn, 1970). Our findings reveal a weak but significant positive relationship between precipitation and the mean leaf area across the study sites, suggesting that precipitation may be a driver of leaf area in local communities. However, in our study, vegetation type, regeneration strategy, and growth form had a more pronounced influence on shaping the leaf area trait. Fire is another selective force for Mediterranean species operating as fire regimes at the local scale. Although variability in regeneration traits can be attributed to fire regimes in the Mediterranean Basin (Moreira et al., 2012), fire‐related traits could not be expected to explain much variability in leaf traits. In our study, in contrast, we found that resprouting ability and regeneration strategy account for some variability in leaf traits. Physiological differences between the two regeneration syndromes also lead to the coexistence of these two regeneration strategies in Mediterranean vegetation (Vilagrosa et al., 2014). Such differences are attributed to a trade‐off between drought resistance and carbon storage (Paula & Pausas, 2006) since resprouters have to allocate more resources to their roots and underground organs that allow them to resprout after a fire. Consequently, resprouters are less tolerant to drought than non‐resprouters in the Mediterranean Basin as they have higher SLA than non‐resprouters. Moreover, the significant weight of the phylogenetic constraint in the association between the resprouting ability and SLA suggests that long‐term evolutionary processes have also contributed to the differences in SLA values between resprouters and nonresprouters. Although the distinction between resprouters and non‐resprouters explains a significant amount of leaf trait variability in Mediterranean plants (this study; Paula & Pausas, 2006), our results suggest that further functional separation in regeneration mode, by considering the propagule‐persistence trait and the seed bank locality (soil or canopy) of non‐resprouter species, offers a more compherensive explanation for leaf trait variability. Therefore, using multiple regenerative traits, such as regeneration strategy, seed bank location, and fire‐stimuated germination, may enhance our understanding of how trait variability is shaped in plant communities of the Mediterranean Basin (e.g. Huerta et al., 2021; Tavşanoğlu & Gürkan, 2014).

Although leaf traits such as SLA and leaf size do not differ even between woody species and herbs at the global level (Díaz et al., 2016), we found that a considerable portion of the variation in leaf traits can be attributed to the woody growth form. A similar result was obtained by Navarro and Hidalgo‐Triana (2021) for SLA by considering trees, large shrubs, and shrubs in a series of Mediterranean shrublands. These results suggest that structural differences in woody plant forms are responsible for at least some of the observed variability in leaf traits in Mediterranean plants. Vegetation type, growth form, and regeneration mode all contribute to the leaf trait variability in local plant communities of the Mediterranean Basin. The difference in leaf traits among vegetation types considered in our study may also be attributed to the functional distinctness of these five vegetation types (Tüfekcioğlu & Tavşanoğlu, 2022). Besides the variability at functional group and vegetation type levels, we also observed a substantial variation in leaf traits in plant communities at the transect scale. Indeed, there is growing evidence that consistent relationships among leaf economic spectrum traits at the global scale (Wright et al., 2004) may not be expressed at the community scale and leaf trait dimensions can be locally variable (Messier et al., 2017). Consequently, overlooking leaf trait variability at the local scale could underestimate the role of microhabitat filters in community assembly, potentially leading to ineffective ecological decisions, such as those in restoration plans (Shi et al., 2018). Therefore, studying leaf traits in plant communities at the local scale will provide a more compreherensive understanding of leaf trait variability.

## AUTHOR CONTRIBUTIONS

**İrem Tüfekcioğlu:** Conceptualization (supporting); data curation (lead); formal analysis (equal); funding acquisition (lead); investigation (lead); methodology (supporting); project administration (equal); writing – original draft (lead); writing – review and editing (equal). **Çağatay Tavşanoğlu:** Conceptualization (lead); formal analysis (equal); funding acquisition (supporting); investigation (supporting); methodology (lead); project administration (equal); supervision (lead); writing – review and editing (equal).

## CONFLICT OF INTEREST STATEMENT

The authors declare no competing interests.

## Supporting information


Appendix S1.

## Data Availability

The main data associated with analyses in this paper are presented as Appendix S1. Further detailed data are available from authors upon reasonable request.
