# Supplementary material for: Growth form, regeneration mode, and vegetation type explain leaf trait variability at the species and community levels in Mediterranean woody vegetation
Source: Ecol Evol. 2024 Mar 11;14(3):e11145. doi: 10.1002/ece3.11145 (PMC10927360; doi:10.1002/ece3.11145)
Supplement: Supplementary file 1 — Appendix S1. [file ECE3-14-e11145-s001.pdf]

Growth form, regeneration mode, and vegetation type explain leaf trait variability at the species and community levels in Mediterranean woody vegetation

Irem Tüfekcioğlu, Çağatay Tavşanoğlu

Supplementary Material

Table S1. The species whose leaf samples were collected in the study, and their taxonomic status, the number of individuals sampled, and functional groups. Regeneration strategy (*sensu* Pausas 1999; Pausas et al. 2004) includes information on both resprouting ability after the fire (resprouters: *R*+ or non-resprouters: *R*-), post-fire persistence ability via any propagule (propagule-persister: *P*+ or propagule-non-persister: *P*-), and the seed bank locality in propagule-persisters (canopy seed bank: *c* or soil seed bank: *s*). Growth form, resprouting ability, post-fire persistence via propagules, and seed bank locality information are based on the BROT database (Tavşanoğlu and Pausas 2018) and field observations. Nomenclature follows Davis (1965-1985), but taxon and family names were updated according to The Plant List (2013).

**Species**	**Species Code**	**Family**	**No. individuals**	**Growth Form**	**Regeneration Strategy**	**Resprouting Ability**
Arbutus andrachne L.	AAN	Ericaceae	25	large shrub	R+P-	yes
Arbutus unedo L.	AUN	Ericaceae	4	large shrub	R+P-	yes
Calicotome villosa (Poir.) Link	CVI	Leguminosae	2	shrub	R+P+	yes
Ceratonia siliqua L.	CSI	Leguminosae	24	tree	R+P-	yes
Cistus creticus L.	CCR	Cistaceae	54	shrub	R-P+s	no
Cistus parviflorus Lam.	CPA	Cistaceae	11	shrub	R-P+s	no
Cistus salviifolius L.	CSA	Cistaceae	63	shrub	R-P+s	no
Cotinus coggygria Scop.	CCO	Anacardiaceae	5	large shrub	R+P-	yes
Cupressus sempervirens L.	CSE	Cupressaceae	4	tree	R-P+c	no
Cytisopsis pseudocytisus (Boiss.) Fertig	CPS	Leguminosae	5	subshrub	R-P+s	no
Daphne gnidioides Jaub. & Spach	DGN	Thymelaeaceae	20	shrub	unknown	unknown
Daphne sericea Vahl	DSE	Thymelaeaceae	2	shrub	unknown	variable
Erica manipuliflora Salisb.	EMA	Ericaceae	30	shrub	R+P+	yes
Hypericum empetrifolium Willd.	HEM	Hypericaceae	20	subshrub	R+P+	unknown
Juniperus oxycedrus L.	JOX	Cupressaceae	3	large shrub	R+P-	yes
Laurus nobilis L.	LNO	Lauraceae	16	large shrub	R+P-	yes
Lavandula stoechas L.	LST	Lamiaceae	48	subshrub	R-P+s	no
Myrtus communis L.	MCO	Myrtaceae	13	large shrub	R+P-	yes
Olea europaea L.	OEU	Oleaceae	50	tree	R+P-	yes
Osyris alba L.	OAL	Santalaceae	28	large shrub	R+P-	yes

Paliurus spina-christi Mill.	PSP	Rhamnaceae	11	large shrub	R+P-	yes
Phillyrea latifolia L.	PLA	Oleaceae	60	large shrub	R+P-	yes
Phlomis grandiflora H. S. Thomps.	PGR	Lamiaceae	10	shrub	unknown	unknown
Phlomis lycia D. Don	PLY	Lamiaceae	30	shrub	unknown	yes
Pinus brutia Ten.	PBR	Pinaceae	57	tree	R-P+c	no
Pistacia lentiscus L.	PLE	Anacardiaceae	46	large shrub	R+P-	yes
Pistacia terebinthus L.	PTE	Anacardiaceae	14	large shrub	R+P-	yes
Ptilostemon chamaepeuce (L.) Less.	PCH	Compositae	22	shrub	unknown	yes
Pyrus elaeagnifolia Pall.	PEL	Rosaceae	22	tree	unknown	unknown
Quercus aucheri Jaub. & Spach	QAU	Fagaceae	25	large shrub	R+P-	yes
Quercus coccifera L.	QCO	Fagaceae	55	large shrub	R+P-	yes
Quercus infectoria subsp. *veneris* (A.Kern.) Meikle	QIN	Fagaceae	16	large shrub	R+P-	yes
Quercus ithaburensis Decne.	QIT	Fagaceae	1	tree	R+P-	yes
Rhamnus punctata Boiss.	RPU	Rhamnaceae	4	large shrub	unknown	unknown
Ruscus aculeatus L.	RAC	Asparagaceae	5	subshrub	R+P-	yes
Smilax aspera L.	SAS	Smilacaceae	19	liana	R+P-	yes
Styrax officinalis L.	SOF	Styracaceae	18	large shrub	R+P-	yes
Thymbra capitata (L.) Cav.	TCA	Lamiaceae	15	subshrub	unknown	variable

References

Davis PH (eds) (1965–1985) Flora of Turkey and the East Aegean Islands, Volumes 1–9, Edinburg University Press, Edinburgh

Pausas JG (1999) Response of plant functional types to changes in the fire regime in Mediterranean ecosystems: a simulation approach. Journal of Vegetation Science 10:717–722 doi: 10.2307/3237086

Pausas JG, Bradstock RA, Keith DA, Keeley JE, Fire Network GCTE (2004) Plant functional traits in relation to fire in crown-fire ecosystems. Ecology 85:1085–1100 doi: 10.1890/02-4094

Tavşanoğlu Ç, Pausas JG (2018) A functional trait database for Mediterranean Basin plants. Scientific Data 5:180135 doi: 10.1038/sdata.2018.135

The Plant List (2013) The Plant List, version 1.1. http://www.theplantlist.org/ [accessed on 08.12.2020]

Table S2. Minimum, mean, and maximum value of individuals for each species for each leaf trait included in the study. Only species with at least five sampled individuals are included. Species codes are given in Table S1.

Species code	SLA ($mm^2\ mg^{-1}$)			Leaf Thickness (mm)			Leaf Area (mm^2)		
	**min**	**mean**	**max**	**min**	**mean**	**max**	**min**	**mean**	**max**
AAN	3.86	9.79	15.71	0.26	0.31	0.36	904.00	2066.65	3229.30
CCO	11.62	17.79	23.95	0.22	0.27	0.33	1001.10	1172.75	1344.40
CCR	3.67	9.84	16.00	0.25	0.45	0.65	35.80	243.50	451.20
CPA	3.76	5.21	6.66	0.49	0.59	0.70	63.20	193.95	324.70
CPS	7.67	9.42	11.17	0.24	0.33	0.42	26.90	30.45	34.00
CSA	3.64	7.25	10.85	0.21	0.46	0.70	45.50	257.15	468.80
CSI	6.00	9.56	13.12	0.24	0.36	0.47	765.40	1738.80	2712.20
DGN	7.13	15.20	23.26	0.17	0.25	0.33	39.20	93.30	147.40
EMA	3.21	5.66	8.10	0.20	0.38	0.56	3.40	8.15	12.90
HEM	3.60	6.20	8.80	0.21	0.31	0.42	5.40	11.90	18.40
LNO	4.43	8.62	12.80	0.23	0.32	0.40	920.20	2316.05	3711.90
LST	2.21	8.67	15.13	0.23	0.31	0.39	2.20	32.85	63.50
MCO	7.68	13.75	19.82	0.21	0.28	0.36	164.70	379.65	594.60
OAL	4.31	9.99	15.67	0.18	0.39	0.59	13.50	41.10	68.70
OEU	3.23	6.38	9.53	0.28	0.43	0.59	35.10	281.35	527.60
PBR	4.42	7.03	9.64	0.45	0.62	0.80	61.00	143.05	225.10
PCH	2.84	5.43	8.01	0.26	0.40	0.53	46.20	119.65	193.10
PEL	2.76	10.52	18.28	0.17	0.31	0.44	79.90	422.85	765.80
PGR	3.99	6.28	8.56	0.47	0.67	0.86	213.50	743.00	1272.50
PLA	5.11	13.65	22.18	0.20	0.28	0.36	66.90	270.95	475.00
PLE	3.71	7.51	11.31	0.27	0.43	0.59	150.30	313.70	477.10
PLY	3.14	7.36	11.57	0.45	0.87	1.28	133.10	408.15	683.20
PSP	13.11	24.47	35.82	0.14	0.18	0.22	210.60	415.55	620.50
PTE	9.48	14.27	19.06	0.17	0.21	0.26	339.30	918.10	1496.90
QAU	4.86	8.77	12.68	0.29	0.36	0.44	128.30	409.80	691.30
QCO	3.34	10.39	17.43	0.26	0.41	0.56	91.90	321.20	550.50
QIN	7.89	14.08	20.27	0.24	0.30	0.36	309.30	852.95	1396.60
RAC	11.14	13.32	15.49	0.23	0.27	0.31	90.60	136.70	182.80
SAS	10.38	20.53	30.68	0.20	0.25	0.31	583.40	1985.20	3387.00
SOF	11.16	19.39	27.61	0.17	0.23	0.29	395.60	1398.10	2400.60
TCA	5.63	7.54	9.45	0.30	0.45	0.59	17.10	29.70	42.30

Table S3. The results of pairwise comparisons between functional group classes for growth form, resprouting and regeneration strategies following principal components analysis (PCA) considering three leaf traits.

**Growth Form**	R^2	**P**
Large shrub vs. Liana	0.058	0.001
Large shrub vs. Shrub	0.139	0.001
Large shrub vs. Subshrub	0.377	0.001
Large shrub vs. Tree	0.034	0.001
Liana vs. Shrub	0.201	0.001
Liana vs. Subshrub	0.680	0.001
Liana vs. Tree	0.196	0.001
Shrub vs. Subshrub	0.309	0.001
Shrub vs. Tree	0.068	0.001
Subshrub vs. Tree	0.488	0.001
**Regeneration Strategy**		
R+P- vs. R+P+	0.173	0.001
R+P- vs. R-P+c	0.077	0.001
R+P- vs. R-P+s	0.161	0.001
R+P+ vs. R-P+c	0.801	0.001
R+P+ vs. R-P+s	0.254	0.001
R-P+c vs. R-P+s	0.087	0.001
**Resprouting Ability**		
Yes vs. No	0.120	0.001

Table S4. Specific leaf area, leaf thickness, and leaf area of species for each functional classification (growth form, resprouting ability, and regeneration strategy). Values are the mean and the standard error (in parenthesis). Data units are mm^2mg^{-1} for specific leaf area, mm for leaf thickness, and mm^2 for leaf area. *n* is the number of species for each functional group considered in analyses. *L. ratio* is the likelihood ratio estimated for the statistical comparison between a null model including the species as the random factor and the model with both the random and the fixed factor (i.e., growth form, regeneration strategy, or resprouting ability).

	**Growth Form**					**Linear model**	
**Trait**	**Liana**	**Tree**	**Large shrub**	**Shrub**	**Subshrub**	**L.Ratio**	**P**
**Specific leaf area**	15.0 (1.0)	7.0 (0.2)	9.4 (0.3)	6.8 (0.2)	7.3 (2.2)	11.5	0.0214
**Leaf thickness**	0.26 (0.01)	0.44 (0.01)	0.31 (0.00)	0.45 (0.01)	0.31 (0.01)	14.3	0.0065
**Leaf area**	1255.9 (146.8)	399.2 (44.2)	568.4 (36.3)	152.5 (10.9)	22.6 (3.1)	16.4	0.0025
**n**	1	6	16	10	5		

	**Regeneration Strategy**				**Linear model**	
**Trait**	**R+P-**	**R+P+**	**R-P+c**	**R-P+s**	**L.Ratio**	**P**
**Specific leaf area**	9.1 (0.2)	6.5 (0.5)	6.5 (0.2)	6.6 (0.1)	8.1	0.0449
**Leaf thickness**	0.32 (0.00)	0.32 (0.02)	0.57 (0.01)	0.39 (0.01)	21.9	0.0001
**Leaf area**	586.6 (32.1)	7.9 (1.1)	125.3 (5.5)	93.5 (6.3)	14.2	0.0027
**n**	21	2	2	5		

	**Resprouting Ability**		**Linear model**	
**Trait**	**Yes**	**No**	**L.Ratio**	**P**
**Specific leaf area**	8.6 (0.2)	6.6 (0.1)	5.9	0.015
**Leaf thickness**	0.35 (0.01)	0.44 (0.01)	9.0	0.0026
**Leaf area**	517.3 (28.1)	101.5 (5.0)	5.0	0.0252
**n**	25	7		

Table S5. Recorded number of individuals for each species for each vegetation type and their average leaf trait values used for calculating community-weighted means. Some trait values for some species were obtained from other sources: the BROT database (Tavşanoğlu and Pausas 2018; shown with Φ), Hacettepe University Functional Ecology Lab. data (Aktepe 2021; Coşgun 2022; shown with Ψ) and scientific papers (Elmas and Kutbay 2015; Liakoura et al. 2001; Merchant 1998; Specht 1988) (shown with Ω). Data units are mm^2mg^{-1} for specific leaf area (*SLA*), mm for leaf thickness (*Lt*), and mm^2 for leaf area (*LA*).

**Species**	**Vegetation Type**					**SLA**	**Lt**	**LA**
	**Semi-closed forest**	**Open forest**	**Closed shrubland**	**Open shrubland**	**Scrubland**			
Arbutus andrachne	3	2	113	38	0	7.61	0.312	1752.45
Arbutus unedo	2	0	17	0	0	9.12	0.253	1754.52
Asparagus aphyllus	59	126	148	65	27	-	-	-
Asperula brevifolia	0	0	4	15	0	-	-	-
Calicotome villosa	1	38	29	35	5	15.84	0.231	29.31
Celtis australis	0	5	0	0	0	17.60 Φ	-	100.00
Ceratonia siliqua	2	3	4	4	0	7.54	0.375	1242.80
Cistus creticus	399	544	351	210	351	7.42	0.400	129.56
Cistus parviflorus	0	13	0	248	0	6.12	0.620	146.40
Cistus salviifolius	931	824	473	1048	45	6.36	0.413	112.80
Cotinus coggygria	26	1	0	0	0	16.97	0.260	1170.35
Crataegus monogyna	1	0	0	9	4	14.23 Ψ	-	117.80Ψ
Cupressus sempervirens	0	0	6	0	0	2.45Ψ	0.963Ψ	290.40Ψ
Cytisopsis pseudocytisus	17	37	0	0	0	10.18	0.381	32.09
Daphne gnidioides	6	12	0	111	12	14.14	0.278	86.29
Daphne sericea	1	0	4	0	0	5.46	0.316	340.72
Dittrichia viscosa	0	0	0	1	0	9.82Φ	-	53.36Φ
Erica manipuliflora	369	894	0	84	0	5.99	0.315	6.42
Euphorbia acanthothamnos	0	0	10	38	1	-	-	-
Genista acanthoclada	207	868	154	677	453	13.65 Ψ	0.156	21.10Ψ
Hypericum empetrifolium	2	99	497	242	0	6.76	0.296	11.67
Juniperus oxycedrus	0	1	0	0	0	6.52Ψ	0.540Φ	19.70Ψ
Laurus nobilis	3	0	1	1	0	10.39	0.251	1984.72
Lavandula stoechas	175	125	0	26	29	6.57	0.280	12.43
Myrtus communis	13	4	0	5	0	13.60	0.280	301.64
Olea europaea	20	13	113	71	19	5.88	0.416	204.13
Origanum onites	0	5	1	51	38	8.50Ψ	-	9.50Ψ
Osyris alba	0	19	31	79	0	8.80	0.399	35.17
Paliurus spina-christi	4	27	0	0	0	23.39	0.185	515.51
Phillyrea latifolia	180	244	398	65	19	8.32	0.262	211.86

Phlomis grandiflora	0	0	14	63	0	5.22	0.647	657.14
Phlomis lycia	53	11	4	221	59	6.25	0.620	229.17
Pinus brutia	219	214	25	2	0	6.45	0.551	139.43
Pistacia lentiscus	40	28	91	86	6	5.81	0.428	240.36
Pistacia terebinthus	11	12	7	1	0	13.43	0.202	687.58
Populus nigra	1	0	0	0	0	10.60 Φ	-	100.00
Ptilostemon chamaepeuce	0	6	15	1	9	5.95	0.442	99.01
Pyrus elaeagnifolia	9	0	0	14	31	11.98	0.274	384.41
Quercus aucheri	20	0	22	72	8	6.74	0.365	269.79
Quercus coccifera	97	100	149	227	25	6.64	0.374	249.50
Quercus infectoria subsp. *veneris*	43	41	13	3	0	13.01	0.281	805.01
Quercus ithaburensis	0	0	0	0	1	10.78	0.366	1459.93
Rhamnus punctata	0	7	0	0	7	10.41	0.247	83.27
Rubia tenuifolia	0	0	3	0	0	-	-	-
Ruscus aculeatus	5	11	49	0	0	12.97	0.264	134.70
Sarcopoterium spinosum	135	90	25	887	1755	11.91 Ω	0.288Ω	72.50Ω
Satureja thymbra	0	56	0	6	0	2.95Ψ	0.354Ψ	61.09Ψ
Smilax aspera	19	31	102	2	0	19.92	0.244	1269.30
Spartium junceum	0	0	1	0	0	18.80	-	684.25 Ω
Styrax officinalis	71	2	0	11	0	19.08	0.235	1619.04
Teucrium chamaedrys subsp. *syspirense*	0	0	0	1	0	10.32 Φ	-	30.54Φ
Teucrium polium	0	11	0	6	0	9.84Φ	0.310Φ	13.20Φ
Teucrium sandrasicum	0	2	0	0	0	-	-	-
Thymbra capitata	2	153	1	673	138	7.80	0.427	28.69

References

Aktepe N (2021) Variability of the flammability of plants in Turkish red pine (*Pinus brutia* Ten.) forests at population, species and community levels, and the relationship of this variability with the fire regime. PhD Thesis, Hacettepe University, Institute of Science, Ankara.

Coşgun ZL (2022) Change in functional trait structure of plant communities along an elevational gradient in southwestern Anatolia. Master Thesis, Hacettepe University, Institute of Science, Ankara.

Elmas E, Kutbay HG (2015) Plant functional types in Mediterranean enclaves in Western Black Sea Region of Turkey. Turkish Journal of Botany 39:30–39 doi: 10.3906/bot-1402-45

Liakoura V, Manetas Y, Karabourniotis G (2001) Seasonal fluctuations in the concentration of U.V.-absorbing compounds in the leaves of some Mediterranean plants under field conditions. Physiologia Plantarum 111:491–500 doi: 10.1034/j.1399-3054.2001.1110409.x

Merchant AG (1998) The light and water stress tolerance of two invasive legumes: *Cytisus scoparius* (Scotch broom) and *Spartium junceum* (Spanish broom). PhD Thesis, Faculty of the Virginia Polytechnic Institute and State University, Blacksburg, VA, USA

Specht RL (1988) Climatic control of ecomorphological characters and species richness in Mediterranean ecosystems of Australia. In: Specht RL (eds) Mediterranean-Type Ecosystems. Kluwer, Dordrecht, pp 149-155

Tavşanoğlu Ç, Pausas JG (2018) A functional trait database for Mediterranean Basin plants. Scientific Data 5:180135 doi: 10.1038/sdata.2018.135

Table S6. The results of pairwise comparisons between vegetation types following principal components analysis (PCA) considering three leaf traits.

Vegetation Type	R^2	P
Open shrubland vs. Scrubland	0.234	0.001
Open shrubland vs. Closed shrubland	0.242	0.002
Open shrubland vs. Open forest	0.046	0.144
Open shrubland vs. Semi-closed forest	0.078	0.068
Scrubland vs. Closed shrubland	0.557	0.001
Scrubland vs. Open forest	0.091	0.042
Scrubland vs. Semi-closed forest	0.287	0.002
Closed shrubland vs. Open forest	0.209	0.003
Closed shrubland vs. Semi-closed forest	0.057	0.170
Open forest vs. Semi-closed forest	0.088	0.022

Table S7. Mean community weighted mean values for specific leaf area, leaf thickness, and leaf area of species for each vegetation type. Values in parentheses are the standard error of the mean. Data units are mm^2mg^{-1} for specific leaf area, mm for leaf thickness, and mm^2 for leaf area. *L.ratio* is the likelihood ratio estimated for the statistical comparison between a null model including the transect as the random factor and the model with both the random and the fixed factor (i.e., vegetation types).

Trait	Semi-closed forest	Open forest	Closed shrubland	Open shrubland	Scrubland	Linear model	
						**L.Ratio**	**P**
**Specific leaf area**	8.3 (0.4)	8.7 (0.3)	8.4 (0.3)	8.7 (0.3)	11.2 (0.2)	30.7	<0.0001
**Leaf thickness**	0.36 (0.01)	0.33 (0.04)	0.33 (0.00)	0.37 (0.01)	0.29 (0.01)	31.0	<0.0001
**Leaf area**	235.3 (67.1)	115.2 (16.3)	283.4 (44.1)	130.8 (18.5)	79.7 (3.8)	20.4	<0.0001

Table S8. Distance-based functional diversity indices for each vegetation type based on leaf area, SLA, and leaf thickness.

Vegetation types	Functional Richness	Functional Evenness	Functional Divergence	Functional Dispersion
Semi-closed forest	8.607	0.691	0.759	1.465
Open forest	9.329	0.545	0.693	1.007
Closed shrubland	8.079	0.579	0.719	1.269
Open shrubland	7.134	0.446	0.614	1.083
Scrubland	0.918	0.637	0.687	0.650

Table S9. The outcomes from both phylogenetic general linear modeling and non-phylogenetic linear modeling focusing on the assessment of growth form, resprouting ability, and regeneration strategy in relation to the studied leaf traits. The Akaike Information Criterion (AIC) is employed for model comparison, while σ^2 represents the maximum-likelihood estimate of the variance rate, with the corresponding mean and 95% confidence intervals in parentheses. Additionally, Akaike weights (*w*) are also provided to gauge the relative support for each model.

	**Phylogenetic model**			**Non-phylogenetic model**	
	**AIC**	**σ^2**	**w**	**AIC**	**w**
**Leaf Area**					
Null	87.6	0.008 (0.005-0.012)		83.6	
Growth form	81.9	0.006 (0.003-0.008)	0.945	78.0	0.944
Resprouting ability	89.2	0.007 (0.004-0.010)	0.315	85.5	0.278
Regeneration strategy	84.7	0.006 (0.003-0.008)	0.817	77.7	0.952
**SLA**					
Null	0.1	0.001 (0.001-0.013)		-22.7	
Growth form	8.9	0.001 (0.000-0.001)	0.012	-23.1	0.544
Resprouting ability	-3.4	0.001 (0.000-0.001)	0.850	-22.8	0.512
Regeneration strategy	6.1	0.001 (0.000-0.001)	0.046	-22.6	0.479
**Leaf Thickness**					
Null	0.5	0.001 (0.001-0.001)		-29.5	
Growth form	2.6	0.001 (0.000-0.001)	0.259	-33.0	0.855
Resprouting ability	2.6	0.001 (0.000-0.001)	0.263	-30.8	0.659
Regeneration strategy	-2.7	0.001 (0.000-0.001)	0.835	-38.1	0.987

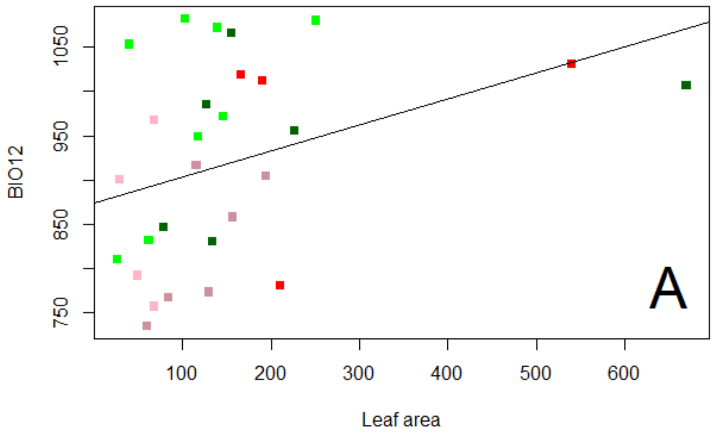

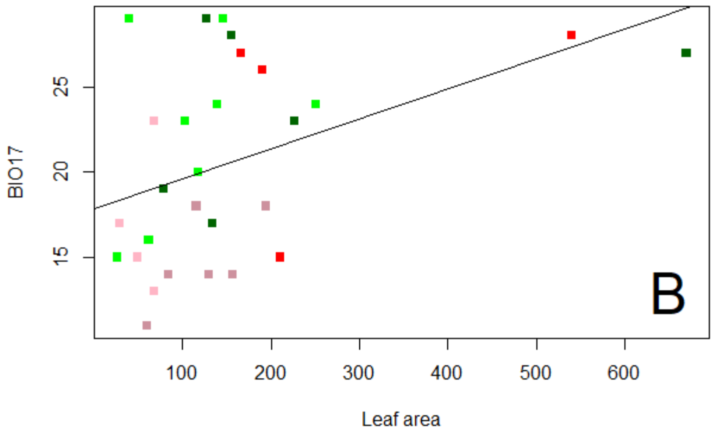

Figure S1. Association between mean leaf area (mm^2) and **(A)** annual total precipitation (mm; BIO12) and **(B)** precipitation of the driest quarter (mm; BIO17). The linear lines represent the results of regression analyses.

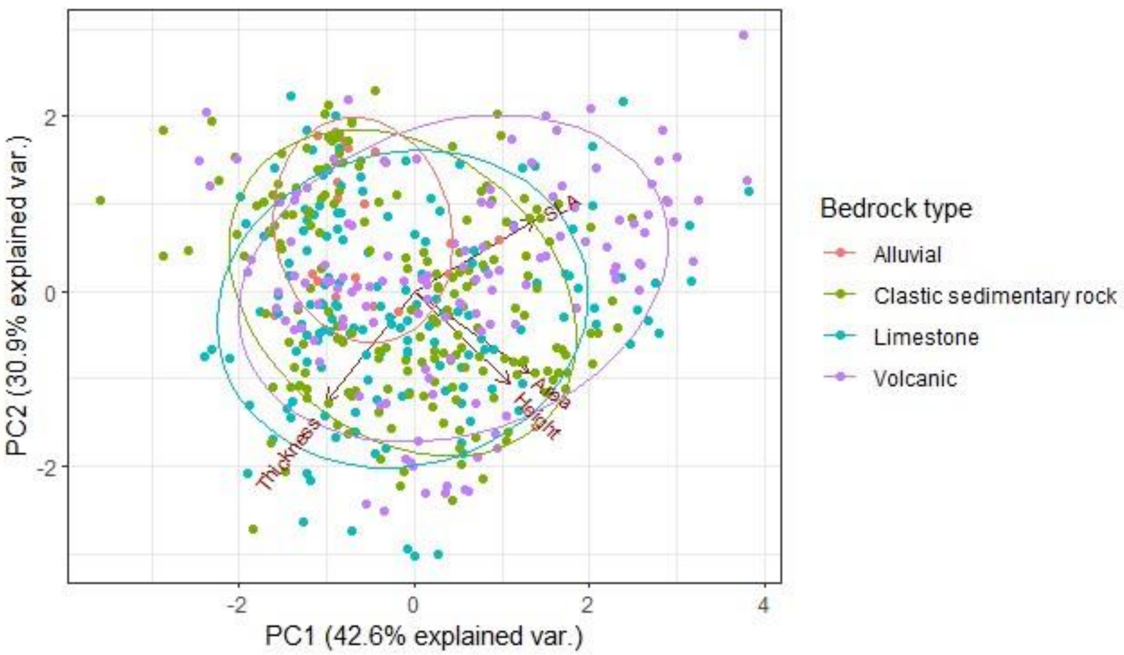

Figure S2. Principal component analysis graph of community weighted mean values for specific leaf area, leaf thickness, and leaf area among different bedrock types. Each data point is community-weighted mean value of each transect, and eclipses indicate the standard deviation of each bedrock type.
